# A lung adenocarcinoma patient with co-mutations of *MET* and *EGFR* exon20 insertion responded to crizotinib

**DOI:** 10.1186/s12920-022-01291-z

**Published:** 2022-06-23

**Authors:** Yan Chen, Bo Jiang, Yuange He, Chu Zhang, Wenjie Zhou, Cheng Fang, Dejian Gu, Minxia Zhang, Mei Ji, Juntao Shi, Xin Yang

**Affiliations:** 1 Department of Oncology, The Third Affiliated Hospital of Soochow University, The First People’s Hospital of Changzhou, No 185 Juqian Road, Tianning District, Changzhou, 213000 China; 2 Department of Thoracic Surgery, The Third Affiliated Hospital of Soochow University, The First People’s Hospital of Changzhou, No 185 Juqian Road, Tianning District, Changzhou, 213000 China; 3 Geneplus-Beijing, 9th Floor, No.6 Building, Peking University Medical Industrial Park, Zhongguancun Life Science Park, Beijing, 102206 China; 4 Department of Cardiothoracic Surgery, The Third Affiliated Hospital of Soochow University, The First People’s Hospital of Changzhou, No 185 Juqian Road, Tianning District, Changzhou, 213000 China

**Keywords:** NCSLC, *MET* mutation, *EGFR* mutation, Double mutation, Crizotinib

## Abstract

**Background:**

Targeted therapy has revolutionized the treatment of patients with malignancies harboring mutations in driver genes and has brought a favorable survival benefit to the population with actionable oncogenic mutations. In recent years, the *MET* exon14 skipping mutation has been recognized as a potentially promising therapeutic target in non-small cell lung cancer (NSCLC). These changes are mutually exclusive with molecular drivers such as *EGFR, KRAS, HER-2*, *BRAF, ALK* and *ROS1*. The prevalence rate of coexisting *MET* exon 14 mutations and *EGFR* sensitive mutations (L858R, exon 19 deletions) in Chinese population was reported to be 0.2% (3/1590). However, the coexistence of *MET* exon 14 mutations with *EGFR* exon 20 insertion mutations has never been reported and the management of this subtype is not identified.

**Case presentation:**

A 69-year-old male with a right lung adenocarcinoma (T4N2M0, IIIB) was confirmed to be positive for *MET* exon 14 skipping (c.3028_3028+1delGGinsTT, 44.4%), *MET* amplification (copy number 4.4), and *EGFR* exon 20 insertion (p. N771_H773dup, 22.1%) mutations. After the progression of one cycle of chemotherapy (Pemetrexed 0.8 g d1), the patient was subsequently accepted treatment with Crizotinib (250 mg twice a day) and achieved an important clinical remission for six months until the development of brain metastases. Then, he was submitted to a cycle of anti-programmed cell death-1 (PD-1) therapy after failure of Crizotinib and eventually acquired resistance despite of the high expression of programmed death ligand-1 (PD-L1) and tumor mutational burden (TMB) status.

**Conclusion:**

This case report provides treatment strategies for epidermal growth factor receptor tyrosine kinase inhibitors (EGFR-TKIs)-untreated lung adenocarcinoma patients simultaneously carrying *MET* alterations and *EGFR* exon 20 insertion mutations. In addition, the signatures of PD-L1 or TMB expression were not the candidate for predicting the efficacy of immunotherapy in this context.

## Background

Lung cancer is the most common cause of cancer-related death worldwide, and important advancements have been achieved for the treatment of non-small cell lung cancer (NSCLC) in recent years. The leading strategies are targeted therapy and immunotherapy with subsets of patients treated according to their genetic aberrations and the expression of programmed death ligand-1 (PD-L1) [1]. Interactions between negative regulatory molecule programmed cell death-1 (PD-1) or its ligand PD-L1 may deliver co-inhibitory signaling to T cell receptors, leading an immunosuppressive microenvironment. PD-1/PD-L1 inhibitors have been an established therapy and achieved unprecedented long-term clinical effect for the capability of restoring the function of T cells to kill tumor cells [2, 3]. Mutations in *EGFR*, *ALK*, and *MET* indicate that patients may experience clinical benefit with the corresponding targeted drugs [4]. In recent years, the multitargeted inhibitor Crizotinib has been approved for patients with *ALK*/*ROS1* rearrangements, *MET* amplification or exon14 skipping mutations [5, 6].

Although the corresponding relationship between targeted drugs and some oncogenic mutations is quite certain, the coexistence of actionable mutations in the same tumor has been little researched, and the treatment of these patients remains unclear. Dysregulation of the *MET* gene frequently occurs as a resistance mechanism to epidermal growth factor receptor tyrosine kinase inhibitors (EGFR-TKIs) therapy. The reported frequency of concomitant *MET* exon 14 mutations and *EGFR* mutations (L858R, exon 19 deletions) in Chinese population was 0.2% (3/1590), representing a rare event in NSCLC. However, the coexistence with *EGFR* exon 20 insertion mutation has never been reported [7]. Here we report the first case of a non-EGFR-TKIs treated patient who harbored both *EGFR* exon 20 insertion and *MET* mutations developed lymph node enlargement after the first single-agent chemotherapy with Pemetrexed for 9 days. Subsequent attempt of Crizotinib treatment resulted in an important 6-month partial remission (PR). However, the patient ultimately failed to two-week PD-1 therapy and delivered extensive systemic metastases despite the high expression of PD-L1 and tumor mutational burden (TMB) status.

## Case presentation

**Fig. 1:**
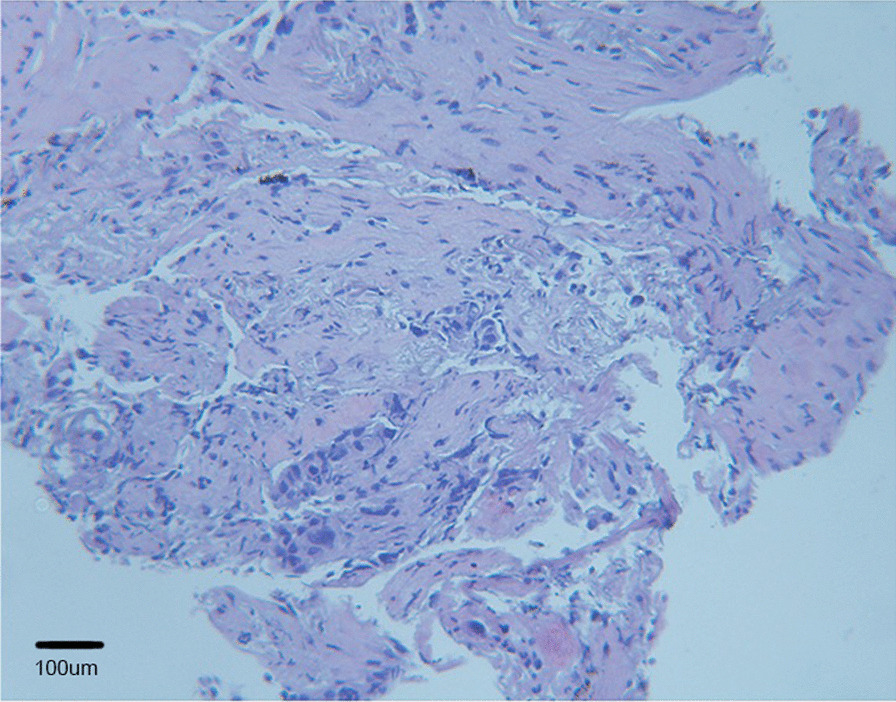
Hematoxylin and eosin staining for lung adenocarcinoma. The sample was formalin-fixed, paraffin-embedded, and then stained with eosin. Hematoxylin and eosin staining (H&E) showed very few atypia cells showing adenoid differentiation (×40). Microscopy was performed using an Olympus BX53microscope (Japan) and Guangzhou Mingmei MD30 camera (China) with no filter, acquisition software was MicroShot basic, Mingmei MD30 cellsens Entry camera system with a resolution of 1000 (W) × 563(H) pixels and no downstream processing. Scale bar: 100 µm

**Fig. 2:**
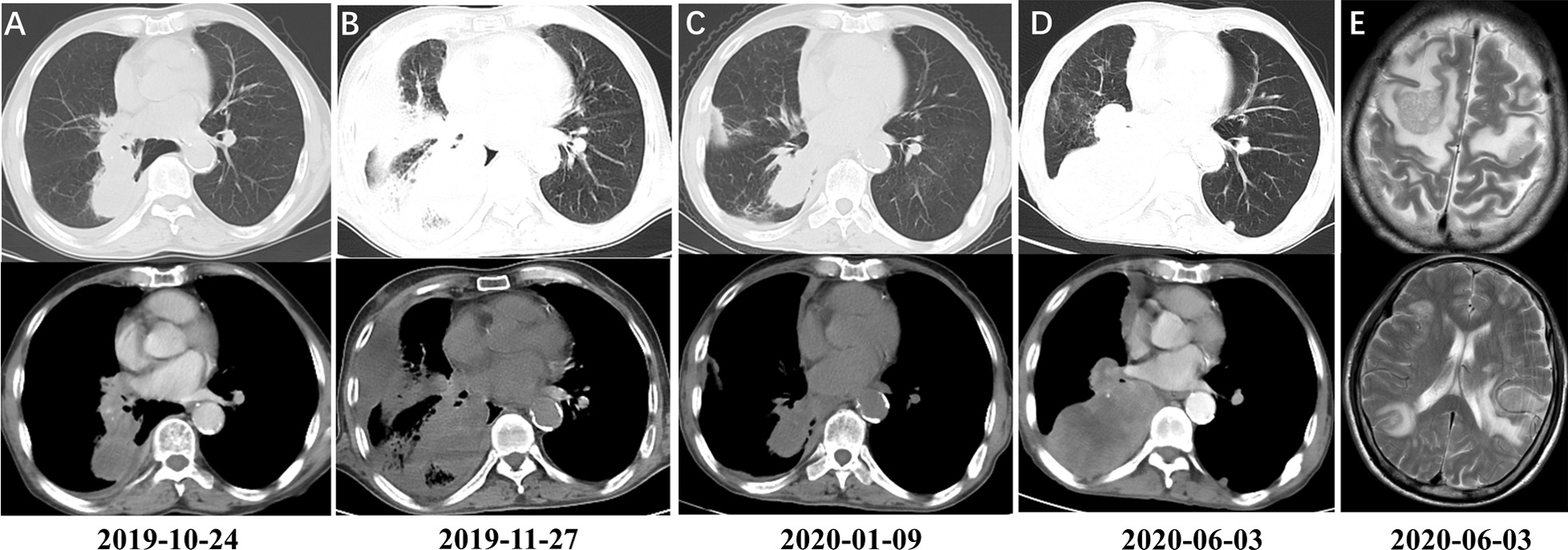
Dynamic imaging of lung lesions at different stages of Crizotinib treatment. **A** The baseline of diagnosis (before treatment): Chest computed tomography scan revealed a mass in the right middle and lower lobe (3.6*3.4 cm), and enlarged lymph nodes in the right hilum and mediastinum; **B** The first evaluation after one cycle of chemotherapy showed progressed of disease with enlarging lymph nodes in the right hilum and mediastinum; **C** The second evaluation after 1 month of Crizotinib therapy showed partial remission according to RECIST1.1 criteria; **D** and **E** Evaluation after approximately 6 months of Crizotinib treatment showed progressive disease of lung lesion and metastasis in right pleura and brain. The measuring scale of all CT image is 20 cm (equipment: GE optima 2), and the MRI is 10 cm (equipment: Philips multiva 1.5 T)

A 69-year-old male patient with a body mass index (BMI) of 18.4(kg/m^2^), presented to the Third Affiliated Hospital of Soochow University in October 2019, complaining of pulmonary lesions for 10 days, detected on radiographic follow-up imaging. The patient had a 40-pack-year smoking history with no family history of cancer. A systematic review of patients found that he had a history of microsatellite stable (MSS) sigmoid cancer (pT3N0M0, stage IIA) with wild-type *KRAS/BRAF* mutations and had undergone partial sigmoid cancer resection combined with mesenteric lymph node dissection in August 2018 without adjuvant chemotherapy after surgery. Postoperative pathology showed moderately differentiated moderately ulcerative adenocarcinoma (3*2.5 cm), invading the full-thickness bowel wall, with no accumulation of upper and lower resection margins, and no cancer metastasis in mesenteric lymph nodes (0/14). Immunohistochemical results showed positive results for PMS2, MSH6, MLH1, MSH2 and Ki67 (50%), as well as negative results for CerbB-2 and P53. Complete remission (CR) was achieved after 13 months of regular follow-up. However, pulmonary lesions was dectected during the follow-up imaging.Pathologic examination of the bronchoscopic biopsy indicated adenocarcinoma (Fig. 1). The chest computed tomography (CT) at baseline revealed a mass in the right middle and lower lobe and enlarged lymph nodes in the right hilum and mediastinum on October 24, 2019 (Fig. 2A). Immunohistochemical staining showed positive results for thyroid transcription factor-1 (TTF-1), novel aspartie proteinase A (Napsin A), Cytokeratin 7 (CK7), cytokeratin (AE1/ AE3) and PD-L1 (approximately 50% of tumor cells), as well as negative results for cytokeratin 5/6 (CK5/6) and P40, confirming a diagnosis of right lung adenocarcinoma (T4N2M0, IIIB) according to the eighth edition of the TNM classification of lung cancer (Fig. 1).

**Fig. 3:**
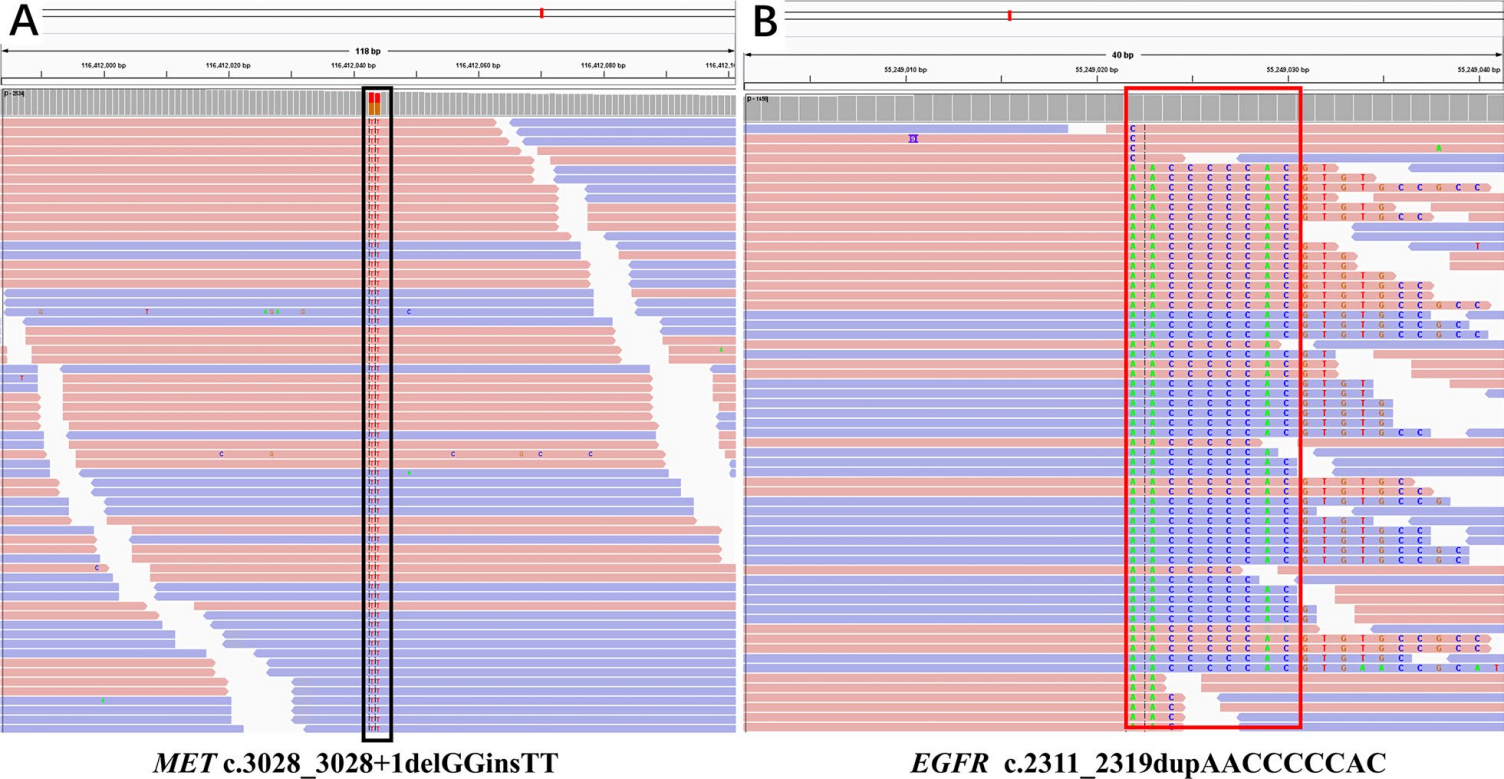
Sequencing reads of MET and EGFR mutations by the Integrative Genomics Viewer. **A** The mutation of *MET* exon14 skipping (c.3028_3028+1delGGinsTT), indicated by the black box; **B** The mutation of *EGFR* exon 20 insertion (c.2311_2319dupAACCCCCAC), indicated by the red box

With informed consent, paired tumor-normal targeted next generation sequencing of 1021 cancer-related genes (Geneplus- Beijing Ltd, Beijing, China) was performed on DNA derived from bronchoscopic biopsy tissue and leukocytes on November 6, 2019. In total 29 somatic mutations were identified, including actionable targets such as *MET* exon14 skipping (c.3028_3028+1delGGinsTT, 44.4%) (Fig. 3A), *MET* amplification (copy number 4.4), *EGFR* exon 20 insertion (p.N771_H773dup, 22.1%) (Fig. 3B), *NF1* IVS41(c.6365-2A>C, 9.3%), and *CCND1* amplification (copy number 3.4). Table 1 lists all of the identified somatic mutations, including single-nucleotide variations, small insertions/deletions, copy number variations, and rearrangements. The TMB and microsatellite instability (MSI) were calculated as previously described [8, 9], and the results were TMB-H (28.2 Muts/Mb>20 Muts/Mb) and MSS.

**Table 1:** List of all identified somatic mutations, including single-nucleotide variations, small insertions/deletions, copy number variations, and rearrangements

Gene	Transcript	Nucleic acid changes	Amino acid changes	Functional area	Frequency/copy number
*MET*	NM_000245.2	c.3028_3028+1delGGinsTT	-	EX14-IVS14	44.4%
*MLL3*	NM_170606.2	c.7151G[2>1]	p.Q2385Rfs*67	EX37	38.4%
*SERPINB4*	NM_002974.2	c.964G>C	p.G322R	EX8	25.7%
*CDC73*	NM_024529.4	c.1590A>T	p.R530S	EX17	24.4%
*EGFR*	NM_005228.3	c.2311_2319dupAACCCCCAC	p.N771_H773dup	EX20	22.1%
*TP53*	NM_000546.5	c.548C>A	p.S183*	EX5	17.9%
*TBX3*	NM_016569.3	c.97GT	p.V33L	EX1	15.9%
*RNF43*	NM_017763.4	c.625A>G	p.I209V	EX6	14.3%
*TP53*	NM_000546.5	c.461G>T	p.G154V	EX5	12.3%
*ATRX*	NM_000489.3	c.5594G>A	p.R1865K	EX23	11.4%
*LRP1B*	NM_018557.2	c.11063T>C	p.L3688P	EX72	10.7%
*NF1*	NM_000267.3	c.6365-2A>C	–	IVS41	9.3%
*BCOR*	NM_001123385.1	c.2965A>T	p.S989C	EX4	9.1%
*KDM5C*	NM_004187.3	c.2953G>C	p.E985Q	EX19	8.6%
*STAT3*	NM_139276.2	c.1002C>A	p.D334E	EX10	8.4%
*TBX3*	NM_016569.3	c.1565C>A	p.P522H	EX7	8.1%
*FANCD2*	NM_033084.3	c.1767-1G>C	-	IVS19	5.0%
*PALB2*	NM_024675.3	c.129G>C	p.K43N	EX3	4.8%
*TP73*	NM_005427.3	c.806G>T	p.R269L	EX7	4.7%
*OR4C6*	NM_001004704.1	c.220G>C	p.V74L	EX1	4.5%
*KDR*	NM_002253.2	c.3625G>T	p.D1209Y	EX27	4.3%
*MLL3*	NM_170606.2	c.14292G>C	p.K4764N	EX55	3.9%
*MLL*	NM_001197104.1	c.3690G>C	p.E1230D	EX7	3.9%
*ABL2*	NM_007314.3	c.125C>T	p.T42I	EX1	3.7%
*ARID1A*	NM_006015.4	c.2369A>G	p.Q790R	EX7	3.5%
*MLL3*	NM_170606.2	c.14035C>T	p.P4679S	EX54	3.5%
*U2AF1*	NM_006758.2	c.235G>A	p.D79N	EX4	3.4%
*CDKN2A*	NM_000077.4	c.164G>T	p.G55V	EX2	3.3%
*MLL3*	NM_170606.2	c.7322G>T	p.G2441V	EX37	3.2%
*NOTCH1*	NM_017617.3	c.4501A>T	p.S1501C	EX25	3.0%
*GRM3*	NM_000840.2	c.1037A>T	p.H346L	EX3	2.6%
*FAT1*	NM_005245.3	c.7421G>T	p.S2474I	EX10	1.9%
*AKT2*	NM_001626.4	Amplification	–	All exon	2.2
*MET*	NM_000245.2	Amplification	–	All exon	2.2
*CCND1*	NM_053056.2	Amplification	–	All exon	1.7

“–” means undetectable. Variant gene frequencies are defned as fractions of variant versus total sequencing read count expressed as percentages

According to the National Comprehensive Cancer Net-work clinical practice guidelines for NSCLC (2019, Version 4), the patient should receive 6 cycles of Pemetrexed (500 mg/kg) and Carboplatin (AUC 6.0 mg/mL/min) administered at 21-day intervals as first-line therapy. The patient refused platinum-based chemotherapy for being informed of the gastrointestinal adverse reactions. Therefore, one cycle of chemotherapy (Pemetrexed monotherapy 0.8 g d1) was first given on November 18, 2019. After the first round of chemotherapy, he developed chest tightness and fatigue symptoms with a poor general condition. Subsequently, the first evaluation by CT showed progressed of disease with enlarged lymph nodes in the right hilum and mediastinum on November 27, 2019 (Fig. 2B). Considering the alteration of *MET* (Exon 14 skipping and amplification), Crizotinib was initiated at a dose of 250 mg twice a day. After 1 month of treatment, a significant decrease in tumor size was achieved with improvements in the patient’s symptoms and functional status. According to the Response Evaluation Criteria In Solid Tumors v1.1, PR was achieved (Fig. 2C).

Approximately six months later (June 2020), the patient readmitted to our hospital suffering from weakness of both upper limbs and back pain. Follow-up CT scan of the chest and abdomen showed progression of the lung lesions with postobstructive pneumonia and metastatic nodules in the right pleura with associated malignant pleural effusions (Fig. 2D). Brain magnetic resonance imaging (MRI) demonstrated multiple brain metastases in bilateral cerebral hemispheres(Fig. 2E). All of these symptoms indicated that the tumor had progressed despite Crizotinib therapy. Considering high level of PD-L1 expression (about 50%), the patient received a third-line treatment of Camrelizumab (200 mg) combined with Bevacizumab (300 mg) in June 2020. During treatment, the patient suffered from fatigue and weight loss. The diseases progressed further after one cycle of combined therapy. On June 16, 2020, whole-brain radiotherapy was administered because of the widespread brain metastases. Informed consent was obtained from the patient prior to treatment. In an attempt to improve tolerance to treatment, palliative intensity modulated radiation therapy (IMRT) was used. The patient received a dose of 30 Gy to the whole brain tissue, 52 Gy to left parietal lesion, and 30 Gy to right parietal lobe, right occipital lobe, and left temporal lobe. However, the patient finally died after two rounds of radiation therapy in July 2020.

## Discussion and conclusions

This case report describes the treatment courses of a lung adenocarcinoma patient with coexisting of *MET* exon14 skipping, *MET* amplification and *EGFR* exon20 insertion mutations. This patient, harboring *MET* alterations and *EGFR* exon 20ins was responded to Crizotinib for approximately 6 months but was resistant to immunotherapy despite the high level expression of PD-L1 and TMB-H status.

To our knowledge, this is the first report of lung adenocarcinoma carrying a double *MET* alterations (exon14 skipping and amplification) and *EGFR* exon 20 insertion mutations in an EGFR-TKIs-untreated patients, if similar cases are encountered in the future, our treatment strategy may have some implications for these patients and their clinicians.

*MET* and *EGFR* are established therapeutic targets in NSCLC. The incidence of *MET* exon 14 skipping mutations in NSCLC is 3–4%, and *EGFR* exon 20 insertion mutations account for about 4–12% of the total *EGFR* mutations [10, 11]. EGFR-TKIs have been the standard option for *EGFR*-sensitizing mutations, such as exon19 deletion, exon21 L858R and other common mutations, and Crizotinib has been recommended for high-level *MET* amplification or *MET* exon 14 skipping mutations [12, 13]. Among substantial *EGFR* exon 20 insertion types, the p.A763_Y76insFQEA mutation (5–6%) displays sensitivity to approved EGFR-TKIs, while the clinical efficacy of other types is extremely limited [14]. As previously studied, *MET* dysregulation is a mechanism of acquired resistance to EGFR-TKIs [15–18]. The coexistence of *MET* amplification with *EGFR* mutations (1.4%) was previously reported in 2012, indicating that the progression-free survival (PFS) of patients with coexisting mutations was significantly shorter than that for patients with *EGFR* mutations alone [19]. Another study included 207 patients with advanced NSCLC and acquired resistance to EGFR-TKIs and suggested that 6.8% (14/207) had coexising *MET* over expression and *EGFR* T790M mutations with a medium post-progression survival time of 10.7 months when treated with EGFR-TKIs plus a MET-tyrosine kinase inhibitors (MET-TKIs) [20]. The most recent study reported a female lung adenocarcinoma patient with the *EGFR* L858R mutation at baseline. The patient progressed after adjuvant Erlotinib therapy for approximately 58 months, subsequent genetic testing at that time was positive for *EGFR* T790M, *MET* amplification and *MET* exon14 mutations (0.23%, 2/866), so the therapy was changed to Osimertinib with MET-TKI (Crizotinib) and achieved a durable clinical response to this combination [21].

Notably, an in-vitro study showed that the expression of *MET* exon14 up-regulated the phosphorylation of *EGFR* and the interaction of *MET* with *EGFR* could drive the activity of the *EGFR* gene, which resulted in a blunting of the inhibition of *EGFR* phosphorylation by EGFR-TKIs. However, *MET* inhibition restored the antagonistic effect of Osimertinib on *EGFR* signaling [21, 22]. This result suggested a complex interaction between *MET* and *EGFR* in NSCLC and provided evidence for potential management strategies that the combination of Osimertinib and Crizotinib may be applicable for *EGFR*/*MET* exon14 co-altered lung cancers. In our case, this patient harbored an *EGFR* exon 20ins mutation, and most of these uncommon mutations may predict resistance to EGFR-TKIs [23, 24]. Therefore, our patient received Crizotinib monotherapy rather than a combination regimen with EGFR-TKIs. The phase II METROS trial demonstrated that the PFS and overall survival (OS) with *MET* amplification or exon14 skipping mutations were 4.4 months (95% CI, 3.0–5.8) and 5.4 months (95% CI, 4.2–6.5) respectively [25]. Encouragingly, the current patient harboring *MET* exon14 skipping, *MET* amplification, and *EGFR* exon20ins achieved approximately 6 months of PR with Crizotinib treatment. Liquid biopsy of 1021 gene panel plasma ctDNA sequencing was assessed at the time of resistance to Crizotinib. The results showed that *MET* exon14 skipping and *EGFR* exon20 still existed, but the *MET* amplification disappeared, which to some extent explained the mechanism of resistance to Crizotinib targeted therapy.

Immune checkpoints inhibitors (ICIs) are critical for maintaining autoimmune tolerance and regulating the duration and extent of immune responses in peripheral tissues. Although the patient showed high expression of PD-L1 and TMB status at baseline, he did not benefit from Camrelizumab and Bevacizumab. As previous reported, positive oncogenic mutations have a negative impact on immunotherapy [26–28], and *EGFR* exon 20ins mutations may be the reason why this patient did not receive benefit from the treatment.

This case report also has some limitations. First of all, the rarity of the case with a rare co-mutation that has never been reported, making it less persuasive. In addition, the patient with low compliance during the treatment course greatly discounted the treatment effect. In recent years, immunotherapy options have been employed for *MET* exon 14 skipping with highly variable results. Although the extent of benefit from immunotherapy is generally not promising for *MET* exon 14 skipping with a shorter survival benefit compared with MET-TKIs. A retrospective analysis from Wong et al. showed moderate efficacy offered by immunotherapy in subsets of patients with *MET* exon 14 skipping, with a disease control rate (DCR) of 70% [29]. However, the patient in our case progressed too fast after immunotherapy, leading the limited duration of immunotherapy effect to be further observed. In a word, the coexistence of *MET* alterations and *EGFR* exon20ins mutation is a very rare event with no standard therapy, and further in-depth research is needed to identify the appropriate treatment for these patients with coexisting driver gene mutations.

## Data Availability

The raw data of sequencing of the patient in this study are not publicly available in order to protect participant confidentiality, but the datasets generated and analysed during the current study are available in the Genome Sequence Archive (GSA) repository (https://ngdc.cncb.ac.cn/gsa-human/s/9NNcO1EN), accession number HRA002387.

## Abbreviations

BMI: Body mass index
CK5/6: Cytokeratin 5/6
CK7: Cytokeratin 7
CT: Computed tomography
CR: Completed remission
DCR: Disease control rate
EGFR-TKIs: Epidermal growth factor receptor tyrosine kinase inhibitors
IMRT: Intensity modulated radiation therapy
MET-TKIs: MET-tyrosine kinase inhibitors
MSI: Microsatellite instability
MRI: Magnetic resonance imaging
MSS: Microsatellite stable
Napsin: Aspartie proteinase A
NSCLC: Non-small cell lung cancer
ORR: Overall response rate
OS: Overall survival
PD: Progressive disease
PD-1: Programmed cell death-1
PD-L1: Programmed death ligand-1
PR: Partial response
PFS: Progression-free survival
ICIs: Immune checkpoints inhibitors
TMB: Tumor mutational burden
TTF-1: Thyroid transcription factor-1
